# Lipid Profile and Oxidative Stress Markers in Wistar Rats following Oral and Repeated Exposure to Fijk Herbal Mixture

**DOI:** 10.1155/2014/876035

**Published:** 2014-10-20

**Authors:** Oluyomi Stephen Adeyemi, Bukola Temitope Orekoya

**Affiliations:** Department of Biological Sciences, Landmark University, Omu-Aran, Kwara State, Nigeria

## Abstract

This study determined the effect of the oral and repeated administration of Fijk herbal mixture on rat biochemical and morphological parameters. Twenty-four Wistar rats were distributed into four groups of 6. Group A served as control and received oral administration of distilled water daily. The experimental groups B, C, and D were daily and orally exposed to Fijk herbal mixture at 15, 30, and 45 mg/kg, respectively. Treatments lasted for 21 days. The rats were sacrificed under mild diethyl ether anesthesia 24 hr after cessation of treatment. The blood and liver samples were collected and used for the biochemical and morphological analyses. Oral exposure to Fijk caused elevated levels of rat plasma ALT, AST, triglycerides, LDL, and MDA. In contrast, rat plasma HDL, GSH, and ALP levels were lowered by Fijk oral exposure. Also, the herbal remedy caused a dose-dependent elevation in the plasma atherogenic index. The histopathology examinations of rat liver sections revealed inimical cellular alterations caused by repeated exposure to Fijk. Study provides evidence that oral and repeated exposure to Fijk in rats raised the atherogenic index and potentiated oxidative stress as well as hepatic injury.

## 1. Introduction

In recent years, the demand for herbal remedies in Nigeria has been on the rise [1]. Yet there are little or no scientific data to support medicinal claims of these herbal formulations. The scenario contributes to fueling safety concerns of herbal remedy consumption in Nigeria.

Herbals are usually sought after for their health benefits and these have become common medicines in many Nigerian homes [2]. It is recognized that herbal remedies prepared from medicinal plants may have several traditional applications especially in developing nations like Nigeria where access to formal healthcare is limited [3]. However, the increasing commercial promotion of herbals necessitate the need for assessment of safety and validity of medicinal claims. In recent past, several studies have demonstrated the toxic effects of indiscriminate use of packaged herbals [1, 4, 5].

Furthermore, the general belief that herbal medicines are natural, effective, and without adverse effects has immensely contributed to the upsurge in the patronage of herbal formulations [6–8]. The popular belief that herbal remedies are without toxic or undesirable effects has however often been proven otherwise [1, 5, 9].

These and other factors form part of several concerns associated with the use of herbal remedies in developing nations. In Nigeria, studies have revealed that the number of Nigerian medicinal plants screened for the validation of biological activity far outweighs the assessment for potential toxic compounds and contaminants [2, 10, 11]. Also, many herbal formulations being promoted and marketed in Nigeria lack scientific data to support acclaimed medicinal benefits and may pose great health risk to the numerous unsuspecting consumers [1].

Fijk is a polyherbal formulation made from various ethnomedicinal plants. It is marketed in Nigeria by Okooloyun Worldwide Nigeria Limited. According to the Fijk leaflet, the herbal is prepared from a mixture of 13 medicinal plants including* Cassia alata, Citrus Medica var acida, Aloe vera, Cassia augustifolia, Cassia siamea, Kyaha sengalensis, Xylopia aethiopica, Gongronema latifolium, Khaya grandifoliola, Morinda lucida, Anthocelesta djalonensis, Citrullus lanatus, *and* aloe barbaris.*

Fijk herbal mixture is acclaimed by its producers to have several medicinal benefits. This must have contributed to the wide patronage and consumption among the Nigerian populace. According to the Fijk label, the herbal remedy is indicated for treatments of several ailments which include obesity, diabetes, sexual improvement, menstruation pains, infertility, high blood pressure, crawling sensation, and malaria, amongst others. There are scientific reports on the medicinal relevance of the individual plant constituents of the Fijk mixture. However, there are no empirical data to support claims of medicinal benefits or otherwise of Fijk. A search through online research libraries including the Cochrane, PubMed, and Google-Scholar revealed the absence of scientific data on Fijk herbal remedy. The scenario makes likely toxic potential from repeated exposure to the Fijk mixture largely unknown. For the safety of public health, it is essential that herbal remedies have available scientific data on safety/toxicity profiles in order to aid informed choices by consumers. This study aimed to determine the effect of oral and repeated exposure to Fijk on rat plasma biochemical indices and liver morphology.

## 2. Materials and Methods

### 2.1. Chemicals and Reagents

All chemicals and reagents used are of analytical grade. Commercial reagent kits for the assay of alkaline phosphatase (ALP), aspartate aminotransferase (AST), alanine aminotransferases (ALT), total cholesterol (TC), total protein (TP), triglyceride (TAG), and high density lipoprotein cholesterol (HDL-C) were as supplied by Randox Diagnostics, Crumlin, UK.

### 2.2. Experimental Animals

Twenty-four male Wistar rats with weight between 190 and 200 g were obtained from the Experimental Animal Farm at the University of Ilorin, Ilorin, Nigeria. The Wistar rats were housed in animal cages in a well-ventilated experimental room. The rats were allowed to acclimatize for a period of 14 days before the commencement of treatments. The rats had free access to standard rat chow and clean water* ad libitum*. Handling of animals was in accordance with relevant institutional and ethical guidelines as approved for scientific study.

### 2.3. Fijk Herbal Mixture

Fijk herbal mixture was a product of Okooloyun Worldwide Limited, Nigeria.

### 2.4. Experimental Design

The animals were randomly distributed into four (4) groups of five. Further details of groupings and treatments are as follows.

 Control: received distilled water.
 Fijk 15 mg/kg: received Fijk at a dosage of 15 mg/kg body weight.
 Fijk 30 mg/kg: received Fijk at a dosage of 30 mg/kg body weight.
 Fijk 45 mg/kg: received Fijk at a dosage of 45 mg/kg body weight.

The dosages were selected based on corresponding therapeutic doses for human consumption as recommended by the herbal producer. Animals were weighed weekly. The treatment was daily and lasted for 21 days.

### 2.5. Necroscopy

At the end of treatments, rats were fasted overnight and sacrificed under anesthesia in slight diethyl ether. The blood samples were collected into EDTA bottles, spun at 5000 g for 10 minutes using a refrigerated centrifuge (Anke TDL-5000B, Shanghai, China) to obtain the plasma, which was subsequently used for the biochemical analysis. The liver tissues were excised, weighed, and fixed in buffered neutral formalin and used for histopathological examinations.

### 2.6. Biochemical Assays

The biochemical indices were determined in rat plasma using a UV/Vis spectrophotometer (Jenway, Staffordshire, United Kingdom) where applicable. The levels of rat plasma total protein (TP), aspartate aminotransferase (AST-EC: 2.6.1.1), alanine aminotransferase (ALT-EC: 2.6.1.2), alkaline phosphatase (ALP-EC: 3.1.3.1), and lipid profile including total cholesterol (TC), triglyceride (TAG), high-density lipoprotein-cholesterol (HDL-C), and low-density lipoprotein cholesterol (LDL-C) were determined using Randox assay kits. Reduced glutathione level (GSH) was determined by the procedure described by Ellman [12] with slight modification. Lipid peroxidation was ascertained by monitoring the degrees of lipid peroxides in the supernatant fraction of plasma using the method described by Niehaus Jr. and Samuelsson [13].

### 2.7. Histopathology Examination

The rat liver was fixed in 10% buffered neutral formalin (BNF) immediately following excision from animals. Fixed tissues were subsequently processed for histopathology examinations as previously described [14]. Capture and scoring for morphological changes were done by a pathologist blind to the treatments, at the Pathology Unit, University of Ilorin Teaching Hospital, Ilorin, Nigeria.

### 2.8. Data Analysis

All data are presented as the mean ± SEM. Data were subjected to statistical analysis using the one-way ANOVA (GraphPad Software Inc., San Diego, CA). Differences among the group means were evaluated by Tukey's test. Mean values were considered to be statistically significant at *P* < 0.05.

## 3. Results

**Figure 1:**
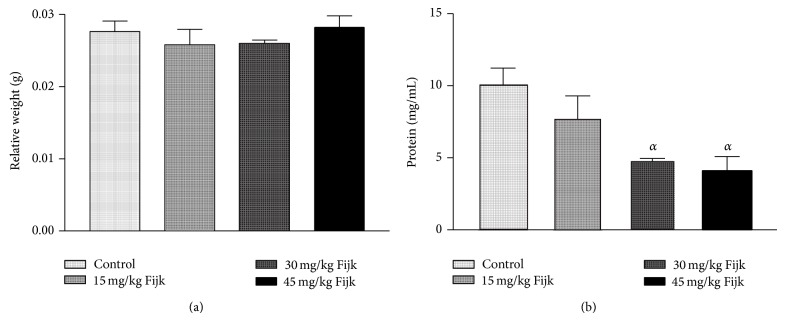
(a) Rat organ-to-body weight ratio following repeated and oral exposure to Fijk herbal at different dosages; (b) level of rat plasma protein following repeated and oral exposure to Fijk herbal at different dosages. Data are presented as mean value ± standard error of mean (SEM), *n* = 6. *α* is significant at *P* < 0.05 relative to control.

**Figure 2:**
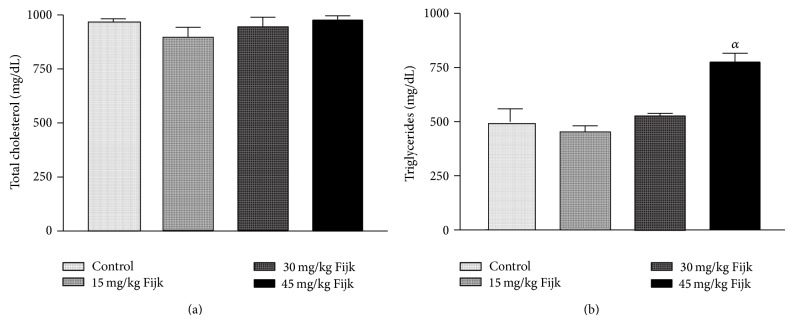
(a) Level of rat plasma total cholesterol following repeated and oral exposure to Fijk herbal at different dosages; (b) level of rat plasma triglyceride following repeated and oral exposure to Fijk herbal at different dosages. Data are presented as mean value ± standard error of mean (SEM), *n* = 6. *α* is significant at *P* < 0.05 relative to control.

**Figure 3:**
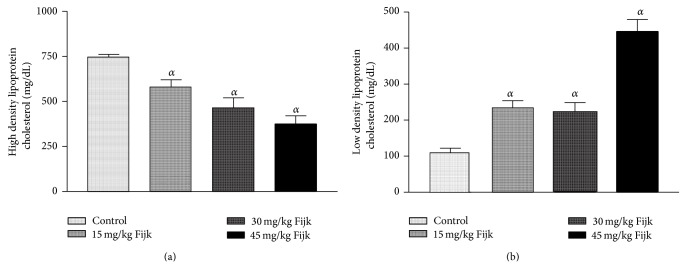
(a) Level of rat plasma high density lipoprotein-cholesterol following repeated and oral exposure to Fijk herbal at different dosages; (b) level of rat plasma low density lipoprotein-cholesterol following repeated and oral exposure to Fijk herbal at different dosages. Data are presented as mean value ± standard error of mean (SEM), *n* = 6. *α* is significant at *P* < 0.05 relative to control.

**Figure 4:**
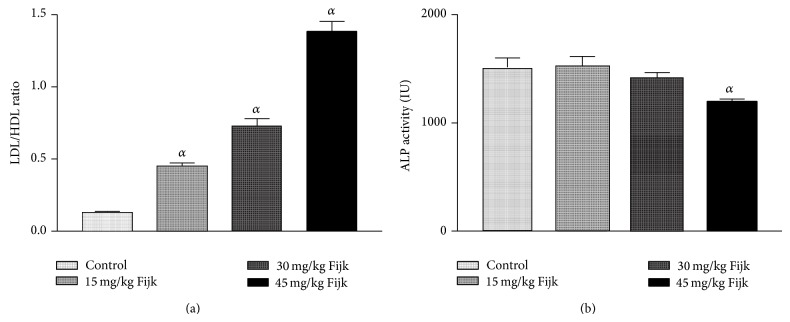
(a) Atherogenic index in rats following repeated and oral exposure to Fijk herbal at different dosages; (b) level of rat plasma alkaline phosphatase following repeated and oral exposure to Fijk herbal at different dosages. Data are presented as mean value ± standard error of mean (SEM), *n* = 6. *α* is significant at *P* < 0.05 relative to control.

**Figure 5:**
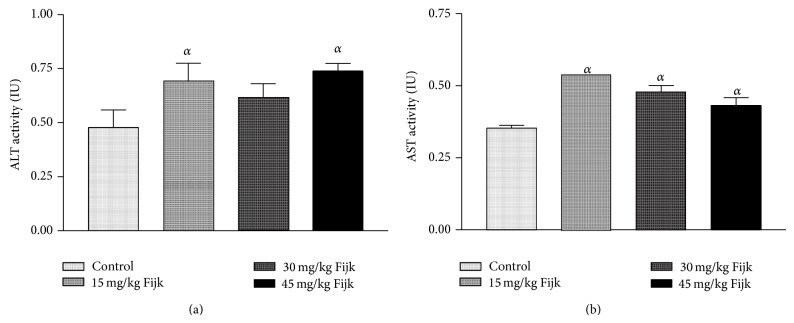
(a) Level of rat plasma alanine transaminase following repeated and oral exposure to Fijk herbal at different dosages; (b) level of rat plasma aspartate transaminase following repeated and oral exposure to Fijk herbal at different dosages. Data are presented as mean value ± standard error of mean (SEM), *n* = 6. *α* is significant at *P* < 0.05 relative to control.

The oral administration of Fijk to rats did not produce significant change to average body weights of animals, even though gradual decreases in rat body weights were observed for groups that received Fijk herbal mixture (Figure 1). Likewise, the relative organ to body weight ratio showed no significant difference among the various treatment groups (Figure 2). However, the oral administration of Fijk herbal mixture at double and triple doses caused significant reduction in rat plasma protein level compared to the control group. The determination of lipid profile showed that the oral exposure of rats to Fijk at the various doses did not produce significant change in the plasma level of total cholesterol when compared to the control. However, the exposure to Fijk at the highest dosage caused significant elevation in rat plasma level of tryglyceride. Further assessment of lipid profile revealed that the daily administration of Fijk mixture depleted rat plasma level of HDL-C (Figure 3). Meanwhile, the level of rat plasma LDL-C rose with increasing doses of Fijk herbal. Furthermore, the atherogenic index was elevated significantly in the groups exposed to Fijk mixture (Figure 4). The elevation in atherogenic index was dose dependent. On the other hand, rat plasma ALP level was lowered (*P* < 0.05) following oral exposure to Fijk herbal mixture but the reduction was only significant in the group that received the highest dose of Fijk. In contrast, treatment of rats with Fijk herbal mixture at all doses significantly increased the levels of plasma ALT and AST relative to the control group (Figure 5).

**Figure 6:**
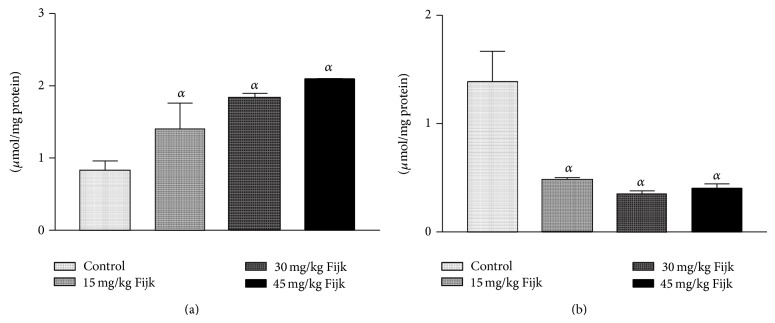
(a) Level of rat plasma malondialdehyde following repeated and oral exposure to Fijk herbal at different dosages; (b) level of rat plasma reduced glutathione following repeated and oral exposure to Fijk herbal at different dosages. Data are presented as mean value ± standard error of mean (SEM), *n* = 6. *α* is significant at *P* < 0.05 relative to control.

To assess whether oral exposure of Fijk in rats would predispose to lipid peroxidation, the plasma level of MDA was determined. There was significant elevation in rat plasma level of MDA in the Fijk treated groups relative to the control (Figure 6). Conversely, oral administration of Fijk to rats significantly depleted the plasma levels of GSH compared to the control.

**Figure 7:**
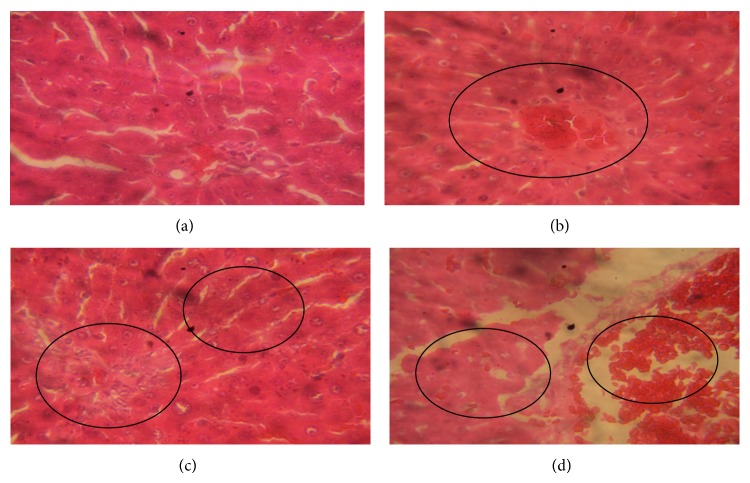
Photomicrographs of rat liver following oral and repeated exposure to Fijk herbal at different dosages. (a) Control group given distilled water showing intact cellular morphology; (b) group given Fijk herbal at 15 mg/kg body weight showing mild inflammation and hemorrhage; (c) group given Fijk herbal at 30 mg/kg body weight showing hyperemia and inflammation; (d) group given Fijk herbal at 45 mg/kg body weight showing hyperemia, ruptured vascular channels and hemorrhage, H&E staining (×400).

The light microscopic examination of rat liver sections for morphological changes revealed lesions caused by Fijk herbal mixture (Figure 7). The Fijk herbal mixture caused hepatic alterations including hypochromic, haemorrahage, ruptured vascular channels, and hyperemia. The severity of lesion increased with increasing doses of Fijk exposure. However, the rat liver in the control group had no incidence of this cellular lession.

## 4. Discussion

There is an increasing demand for herbal products as alternative medicines. Unfortunately the use of herbal products is not strictly regulated in Nigeria thus making them freely available, a scenario which predisposes to possible abuse by consumers [1]. Although herbal mixtures enjoy wide patronage in Nigeria, little is known about likely toxicity that may be associated with repeated consumption. Herbal remedies may have recognizable therapeutic effects; they also may have toxic side effect. More so, many herbal preparations lack scientific facts to back up acclaimed medicinal benefits. Currently, there are no available empirical data on Fijk herbal mixture. The present study sought to determine the influence of oral and repeated adminstration of Fijk herbal mixture on rat biochemical and morphological parameters. This is coming against the background that previous studies have demonstrated toxic potentials of packaged herbals [4, 10, 11].

In the present study, oral adminsitration of Fijk revealed no adverse effect on average rat weight. Although rat liver weights were reduced in the treatment groups, the relative organ weights were not significantly affected by exposure Fijk mixture. However, oral exposure to Fijk decreased rat plasma protein levels. This may implicate reduced synthetic function of the liver. The liver is responsible for the synthesis of most plasma proteins. Studies have associated decreased plasma proteins to impaired liver function [15–17].

The evaluation of rat lipid profile revealed that oral administration of Fijk herbal mixture did not significantly alter the total cholesterol in all the treatment groups. However, there were dose-dependent increases (*P* < 0.05) to the plasma levels of TAG and LDL-C in rats orally exposed to Fijk herbal mixture. Conversely, levels of plasma HDL-C were significantly lowered by repeated administration of Fijk mixture and the effect was dose dependent. The lipid profile indices are useful in monitoring health status of the cardiovascular system. Although the rat total cholesterol level was not affected by Fijk herbal mixture administration, elevated levels of TAG and LDL-C may predispose to cardiovascular related disorders [18]. Increased level of LDL-C has been associated with higher risk of atherosclerosis while elevated level of HDL-C is linked to reduced occurences of cardiovascular disorder [19, 20]. The level of rat plasma HDL-C decreased with increasing doses of Fijk leading to elevated atherogenic index. The atherogenic index can be used to predict the risk for development of cardiovascular disorders. Therefore, the low ratio of HDL-C to LDL-C, caused by repeated oral exposure to Fijk, may implicate increased tendency for the development of atherosclerosis.

The rat plasma levels of ALT and AST were elevated by exposure to repeated administration of Fijk mixture relative to control. The ALT and AST are normally found in the red blood cells, liver, heart, and kidney tissues. The levels of ALT and AST have long been used to assess the functions of liver. Increased plasma levels of both ALT and AST have been linked to tissue toxicity [21, 22]. Normally, a basal level of the enzymes is found in the plasma; however, when there is cellular damage, the enzymes extrude into the extracellular fluid thus raising the concentrations in the plasma. In the present study, the significant alterations to levels of rat plasma ALT and AST may implicate stress imposed on liver by the oral exposure of Fijk. This is further supported by the fact that the ratio of AST to ALT in plasma was less than one (<1), which may indicate ensuing acute or chronic liver injury [23]. On the other hand, the rat plasma ALP levels were significantly reduced in the group which received the highest dosage of Fijk herbal mixture. The decreased level may be as a result of inactivation or decreased protein synthesis [24]. Previous studies have shown that herbal mixtures have capability to alter the levels of ALT, AST, and ALP in rats [1, 9].

Furthermore, in order to evaluate whether the oral and repeated exposure to Fijk mixture was capable of predisposing to oxidative stress, we determined the levels of rat plasma MDA and GSH. The MDA is a by-product of lipid peroxidation while the GSH level could be used to assess the antioxidant status of a cellular system. The increased level of rat plasma MDA suggested the presence of lipid peroxidation caused by oral exposure to Fijk herbal mixture. The increased MDA level in rats may be due to the generation of free radical species potentiated by exposure to Fijk mixture. On the other hand, ensuing oxidative stress caused by the oral administration of Fijk was further demonstrated by the depleted level of rat plasma GSH. The GSH molecule is a nonenzymatic antioxidant capable of scavenging free radicals and it could also serve as substrate to other antioxidant enzymes like the glutathione peroxidase. The low level of GSH may be attributed to increased usage in order to scavenge free radical species or consumption as substrate by antioxidant enzymes which function to protect against oxidative damage [25]. The elevated plasma MDA and low GSH levels are reminiscent of ensuing oxidative stress. Oxidative stress sets in when the levels of prooxidants outweigh the levels of antioxidants. Recent studies have associated oral and repeated exposure to herbal mixtures with lipid peroxidation and oxidative stress [1, 5].

The light microscopic examinations of rat liver sections showed intact and normal cellular architecture in the control group. However, the examination of rat liver sections in groups exposed to various dosages of Fijk mixture revealed incidences of morphological lesions. The rat hepatic alterations caused by exposure to Fijk herbal mixture included haemorrahage, ruptured vascular channels, hyperemia, and inflammation. The histopathological findings support the biochemical alterations caused by oral and repeated exposure to Fijk herbal mixture and are reminiscent of rat hepatic injury. This is consistent with a previous report [10] which demonstrated organ toxicity caused by herbal formulations.

## 5. Conclusion

The study revealed elevated atherogenic index caused by oral and repeated exposure to Fijk mixture in rats. Furthermore, oral and repeated exposure to Fijk herbal mixture in rats caused lipid peroxidation and potentiated hepatic lesion.

## References

[1] Adeyemi O. S., Fambegbe M., Daniyan O. R., Nwajei I. (2012). Yoyo Bitters, a polyherbal formulation influenced some biochemical parameters in Wistar rats. *Journal of Basic and Clinical Physiology and Pharmacology*.

[2] Obi E., Akunyili D. N., Ekpo B., Orisakwe O. E. (2006). Heavy metal hazards of Nigerian herbal remedies. *Science of the Total Environment*.

[3] Adisa R., Fakeye T. (2006). Assessment of the knowledge of community pharmacists regarding common phytopharmaceuticals sold in South Western Nigeria. *Tropical Journal of Pharmaceutical Research*.

[4] Joshua A. J., Goudar K. S., Sameera N., Kumar G. P., Murali B., Dinakar N., Amit A. (2010). Safety assessment of herbal formulations, rumbion and tyrel in Albino Wistar rats. *The American Journal of Pharmacology and Toxicology*.

[5] Oyewo E. B., Adetutu A., Adebisi J. A., Olorunnisola O. S., Adesokan A. A. (2013). Sub-chronic administration of Febi super bitters triggered inflammatory responses in male Wistar rats. *Journal of Medical Sciences*.

[6] Gupta L. M., Raina R. (1998). Side effects of some medicinal plants. *Current Science*.

[7] Kamboj V. P. (2000). Herbal medicine. *Current Science*.

[8] Kashaw V., Amit K. N., Abhinav A. (2011). Hepatoprotective prospective of herbal drugs and their vesicular carriers—a review. *International Journal of Research in Pharmaceutical & Biomedical Sciences*.

[9] Ekor M., Odusoga A. O., Adesina O. O., Adewale G. B., Kolawole S. O. (2010). Toxicity evaluation of Yoyo cleanser bitters and fields Swedish bitters herbal preparations following sub-chronic administration in rats. *The American Journal of Pharmacology and Toxicology*.

[10] Ezejiofor N. A., Onyiaorah V. I., Hussaini D. C., Orisakwe O. E. (2008). Multiple organ toxicity of a Nigerian herbal supplement (U & D sweet bitter) in male Albino rats. *Pakistan Journal of Pharmaceutical Sciences*.

[11] Ogbonnia S. O., Mbaka G. O., Igbokwe N. H., Anyika E. N., Alli P., Nwakakwa N. (2010). Antimicrobial evaluation, acute and subchronic toxicity studies of Leone Bitters, a Nigerian polyherbal formulation, in rodents. *Agriculture and Biology Journal of North America*.

[12] Ellman G. L. (1959). Tissue sulfhydryl groups. *Archives of Biochemistry and Biophysics*.

[13] Niehaus W. G., Samuelsson B. (1968). Formation of malonaldehyde from phospholipid arachidonate during microsomal lipid peroxidation. *European Journal of Biochemistry*.

[14] Adeyemi O. S., Akanji M. A. (2012). *Psidium guajava* leaf extract: effects on rat serum homeostasis and tissue morphology. *Comparative Clinical Pathology*.

[15] Bessesen A., Smith-Kielland A., Gadeholt G., Morland J. (1984). Reduced synthesis of hepatic and plasma proteins in rats during diethyl ether anaesthesia. *Acta Pharmacologica et Toxicologica*.

[16] Adenike S. F., Stephen A. O. (2010). Changes in haematological indices and protein concentrations in *Trypanosoma* brucei infected rats treated with homidium chloride and diminazene aceturate. *EXCLI Journal*.

[17] Adeyemi O. S., Akanji M. A., Ekanem J. T. (2010). Anti-anaemic properties of the ethanolic extracts of *Psidium guajava* in *Trypanosoma brucei* brucei infected rats. *Research Journal of Pharmacology*.

[18] Adeyemi O. S., Akanji M. A. (2011). Iron and nitric oxide play key role in the development of cardiovascular disorder. *Journal of Toxicology and Environmental Health Science*.

[19] Grover-Páez F., Zavalza-Gómez A. B. (2009). Endothelial dysfunction and cardiovascular risk factors. *Diabetes Research and Clinical Practice*.

[20] Olukanni O. D., Akande O. T., Alagbe Y. O., Adeyemi O. S., Olukanni A. T., Daramola G. G. (2013). Lemon juice elevated level of reduced glutathione and improved lipid profile in wistar rats. *American-Eurasian Journal of Agricultural & Environmental Sciences*.

[21] Adeyemi O. S., Akanji M. A. (2011). Biochemical changes in the kidney and liver of rats following administration of ethanolic extract of *Psidium guajava* leaves. *Human and Experimental Toxicology*.

[22] Adeyemi O. S., Sulaiman F. A. (2012). Biochemical and morphological changes in *Trypanosoma brucei* brucei-infected rats treated with homidium chloride and diminazene aceturate. *Journal of Basic and Clinical Physiology and Pharmacology*.

[23] Davern T. J., Scharschmidt B. F., Feldman M., Friedman L. S., Sleisenger M. H. (2002). Biochemical liver tests. *Sleisenger & Fordtran's Gastrointestinal and Liver Disease: Pathophysiology, Diagnosis, Management*.

[24] Malomo S. O., Daramola A. S., Balogun E. A. (1995). Some serum and tissue enzyme changes in mice infected with Plasmodium yoelii nigeriensis before and after administration of halofantrine hydrochloride. *Nigerian Journal of Biochemistry and Molecular Biology*.

[25] Akanji M. A., Adeyemi O. S., Oguntoye S. O., Sulyman F. (2009). *Psidium guajava* extract reduces trypanosomosis associated lipid peroxidation and raises glutathione concentrations in infected animals. *EXCLI Journal*.

